# Clinical, Immunological and Pathological Characteristics of Ischemic Dermatopathy in Dogs with Leishmaniosis

**DOI:** 10.3390/pathogens14030246

**Published:** 2025-03-03

**Authors:** Nuria García, Àlex Cobos, Laia Solano-Gallego, Marina García, Laura Ordeix

**Affiliations:** 1 Fundació Hospital Clínic Veterinari, Facultat de Veterinària, Universitat Autònoma de Barcelona, 08193 Bellaterra, Spain; mgarciaacedos@gmail.com (M.G.); laura.ordeix@uab.cat (L.O.); 2 Departament de Medicina i Cirurgia Animals, Facultat de Veterinària, Universitat Autònoma de Barcelona, 08193 Bellaterra, Spain; laia.solano@uab.cat; 3 IRTA, Animal Health, Centre de Recerca en Sanitat Animal (CReSA), Campus de la Universitat Autònoma de Barcelona, 08193 Bellaterra, Spain; alex.cobos@irta.cat; 4 Unitat Mixta D’investigació IRTA-UAB en Sanitat Animal, Centre de Recerca en Sanitat Animal (CReSA), Campus de la Universitat Autònoma de Barcelona, 08193 Bellaterra, Spain; 5 WOAH Collaborating Center for Research and Control of Emerging and Re-Emerging Pig Diseases (IRTA-CReSA), 08193 Bellaterra, Spain; 6 Departament de Sanitat i Anatomia Animals, Facultat de Veterinària, Universitat Autònoma de Barcelona, 08193 Bellaterra, Spain

**Keywords:** canine, *Leishmania infantum*, diagnosis, vasculopathy, antibodies

## Abstract

Cutaneous lesions suggestive of vasculitis and/or ischemic dermatopathy (ID) are anecdotally reported in canine leishmaniosis, and the clinicopathological features of these conditions have not been fully characterized. The objective of this case series was to describe six dogs with leishmaniosis and ID. In 5/6 dogs, leishmaniosis was diagnosed at the time of ID diagnosis, whereas in 1/6 dogs, ID developed during the first month of anti-*Leishmania* conventional treatment. One each of greyhound, Chihuahua, whippet, American bully, hound and mixed breeds were represented, and the median age at presentation was 6 years [2–8]. All patients presented high or very high levels of circulating anti-*Leishmania infantum* antibodies. The cutaneous lesions were multifocal alopecia with atrophic skin with hyper- or hypopigmentation (6/6), ulcers located on the extremities and trunk (3/6) and onychodystrophy (2/6). Histologically, ID was confirmed by the presence of follicular atrophy (faded follicles) (6/6), perivascular or interstitial lymphoplasmacytic dermatitis or panniculitis (6/6), collagen smudging (3/6), dermal fibrosis (3/6), lymphocytic interface dermatitis (3/6) and ulceration (3/6). Vasculopathy was observed in the superficial and mid-vascular plexuses in 4/6 dogs and characterized by the combination of some of the following lesions: vasocongestion, hemorrhagic foci, mild hyaline mural degeneration, thrombi and fragmented degenerating nuclear debris of neutrophils in the vascular wall. Moreover, myositis was observed in 1/6 cases. *Leishmania*-specific immunohistochemistry was positive in the skin of 4/6 cases. Leishmaniosis might be considered an underlying cause of ID in dogs. However, the immune mechanisms and pathogenesis need to be elucidated.

## 1. Introduction

Ischemic dermatopathies (IDs) are a group of immune-mediated vascular diseases that share clinical and histopathological characteristics despite having different etiologies. Generally speaking, the clinical picture occurs due to a decreased adequate supply of oxygenated blood through the damaged vessels, resulting in ischemia and the subsequent prolonged atrophy of the recipient cutaneous tissue [1]. The exact pathogenesis is unknown; however, cell-poor vasculitis in small-sized cutaneous vessels is suspected, secondary to the deposition of immune complexes that are formed in immune-mediated or infectious diseases or during drug reactions or induced due to vaccination, among others [2].

Clinical signs may include alopecia, scaling, hyper- or hypopigmentation, ulcers and crusts. These lesions are typically found in the marginal areas of the body, such as margins of the pinnae and/or the tip of the tail and/or bony prominences and/or digits/paw pads, because in those regional areas, the vessels are of small caliber [3]. The most common histopathological features described in the literature are adnexal atrophy, cell-poor interface dermatitis, subepidermal collagen smudging, vasculitis or vasculopathy and lymphoplasmacytic dermatitis and/or panniculitis [4].

Classically, IDs have been classified based on clinical and historical features into five subtypes: canine familial dermatomyositis, juvenile onset dermatomyositis-like disease in atypical breeds, post-rabies vaccine vasculitis or panniculitis, generalized vaccine-associated ischemic dermatopathy and generalized idiopathic ischemic dermatopathy [5].

Canine leishmaniosis (CanL) due to *Leishmania infantum* is clinically very pleomorphic. Cutaneous manifestations of CanL can be classified as either typical or atypical [6]. Typical clinical patterns such as scaling dermatitis, ulcerative dermatitis located on bony prominences, papular dermatitis and onychogryphosis are frequent and/or highly indicative of the disease, whereas atypical clinical patterns such as pustular dermatitis, nodular dermatitis, ulcerative dermatitis not located on bony prominences and footpads and/or nasal hyperkeratosis are less frequent and/or can mimic various other diseases, making them less suggestive of CanL. Among the atypical forms of CanL, there are those cutaneous manifestations that suggest vascular damage, such as ulcerative dermatitis located on the margins of the pinnae, the tip of the tail, the digits, and the paw pads [7]. Although rarely documented, the pathogenesis of the ulcers is attributed to cutaneous vasculitis with deposition of circulating immune complexes (CIC) in the vessel wall [8]. In fact, it is suggested that in dogs with leishmaniosis, a marked non-protective production of antibodies, the deposition of CIC in small-caliber vessels’ walls may occur, resulting in clinical signs associated not only with vasculitis but also with uveitis, glomerulonephritis and/or arthritis [9].

Anecdotally, signs of ID may also be observed in dogs with leishmaniosis, and to the best knowledge of the authors, it is important to highlight that the clinical characteristics of dogs with leishmaniosis and ID have never been described. Therefore, the objective of this descriptive clinical study was to describe the clinical, immunological and pathological characteristics of ID in a cohort of dogs with clinical leishmaniosis.

## 2. Materials and Methods

### 2.1. Case Selection

The database of the Dermatology Service of Veterinary Teaching Hospital (Universitat Autònoma de Barcelona, Spain) was thoroughly searched for dogs diagnosed with leishmaniosis and ID between 2013 and 2024. The inclusion criteria were: (1) diagnosis of leishmaniosis based on the presence of compatible clinical signs and/or clinicopathological abnormalities and high levels of specific anti-*L. infantum* antibodies, with or without identification of *Leishmania* amastigotes in cutaneous cytology and/or histology with or without immunochemistry for *Leishmania* [10]; (2) skin lesions indicative of ID as described previously, encompassing multifocal alopecic areas characterized by cutaneous atrophy, scaling and hypo- or hyperpigmentation distributed mainly on the head and distal extremities; (3) histopathological findings compatible with ID; (4) a negative result for the detection of *Dirofilaria immitis* antigen and antibodies to *Anaplasma phagocytophilum*, *Anaplasma platys*, *Borrelia burgdorferi*, *Ehrlichia canis* and *Ehrlichia ewingii* antigens (SNAP 4Dx™); and (5) at least one month of clinical follow-up after anti-*Leishmania* conventional treatment of leishmaniosis.

### 2.2. Data and Sample Collection

Clinical reports were evaluated and signalment (breed, age and sex), age at diagnosis, time of lesion onset, clinical signs, clinicopathological and serological results, treatment and outcome were recorded.

In all dogs, 5–6 mL of blood was taken aseptically by jugular or cephalic venipuncture and was divided into one plain and one EDTA tube for routine laboratory tests (complete blood count, complete biochemistry profile and serum electrophoresis) and serological testing. *Leishmania* serological testing was carried out by a quantitative in-house ELISA. Urine samples were collected by free catch, cystocentesis or catheterism to perform a complete urinalysis and urinary protein creatinine ratio.

### 2.3. Histopathology Evaluation

Skin sections of formalin-fixed, paraffin-embedded cutaneous biopsies that were routinely stained with hematoxylin and eosin were reviewed. The presence of adnexal atrophy (“faded follicles”), cell-poor interface dermatitis, lymphoplasmacytic dermatitis panniculitis and myositis and collagen smudging were recorded. Moreover, histological lesions suggestive of vascular damage such as loss of vascular distinction or endothelial cells, microhemorrhage, perivasal edema, hyaline degeneration and/or the presence of inflammatory cells or nuclear dust in the vascular wall were also noted.

Skin sections were also stained with periodic acid–Schiff (PAS) and phosphotungstic acid-haematoxylin stain (PTAH) to evaluate hyaline vascular changes and vascular fibrin deposition, respectively. Alcian blue stain was also performed to assess mucin deposition and collagen replacement.

### 2.4. Leishmania Infantum Immunohistochemistry

In all cases, the presence of *Leishmania* spp. in dermal infiltrate was evaluated using a standard protocol with an indirect immunoperoxidase method. Haemosiderin pigment was eliminated with 5 percent oxalic acid and amastigotes of *Leishmania* appeared as dark brown-stained bodies, which contrasted with the haematoxylin-stained host cells [11]. Where present, parasite location was defined, and parasite load was determined in positive immunolabelled sections as the average number of microorganisms counted in 10 consecutive high-power fields (400×) of areas with inflammatory infiltrate and interpreted as follows: (a) no microorganisms (negative), (b) 1–10 microorganisms (low) and (c) 11–30 microorganisms (high).

### 2.5. IgG Immunohistochemistry

Additional serial sections were obtained and processed using BOND RX immunostainer to detect IgG within tissues. Briefly, antigen retrieval was applied at 100 °C for 10 min, followed by peroxide block, incubation with rabbit anti-IgG Fc antibody (AB_2868356, Invitrogen, Thermo Fisher Scientific, Waltham, MA, USA) diluted at 1/250.000 for 60 min, followed by Bond Polymer Refine Detection (DS9800, Leica Biosystems, Barcelona, Spain). A dog lymph node was used as a positive control, and unaffected dog skin was used as a negative control. The slides were evaluated using a light microscope, and staining within the vessels was assessed.

## 3. Results

### 3.1. Clinicopathological Data and Treatment Response

**Figure 1:**
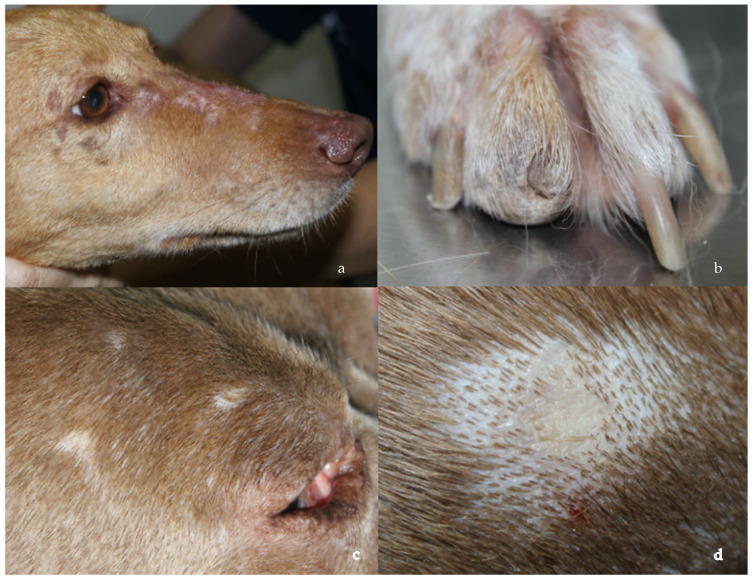
Clinical presentations: (**a**,**c**) multifocal alopecia in the face, (**b**) onychodystrophy and (**d**) atrophic skin.

In the database between 2013 and 2024, six dogs were included in the study, and the breeds present were greyhound, Chihuahua, whippet, American bully, hound and mixed breed. The median age at presentation was 6 years [2–8 years], and the dogs were mostly males (n = 4). In the physical examination, three dogs presented mild generalized lymphadenomegaly, and the skin lesions were multifocal alopecia with atrophic skin with hyper- or hypopigmentation (Figure 1a,c,d) (5/6), ulcers (3/6) and onychodystrophy (Figure 1b) (2/6) (Table S1). These lesions were limited to the face, trunk and to the extremities (including nails) and developed at the time of diagnosis of leishmaniosis (5/6) or during the first month after diagnosis and during treatment of leishmaniosis (1/6).

Bloodwork was performed in all patients before performing skin biopsies and urinalysis, with urinary protein creatinine ratio (UPC) being performed in 3/6 dogs. Mild nonregenerative anemia was present in 2/6 dogs, hypergammaglobulinemia was observed in 5/6 dogs and only one patient of three that, where examined, presented proteinuria with a UPC of 0.8. In relation to anti-*L. infantum* antibodies, five dogs had high results, and one dog had very high antibody levels.

Five of the six cases demonstrated good clinical responses to treatment with meglumine antimoniate (100 mg/kg/day for 28 days) and allopurinol (10 mg/kg/twice a day). These dogs were characterized by healing of erosive–ulcerative lesions and/or hair regrowth after one month of treatment. However, one dog did not respond to anti-*Leishmania* treatment during the 3 months of follow-up.

### 3.2. Histopathological Evaluation

**Figure 2:**
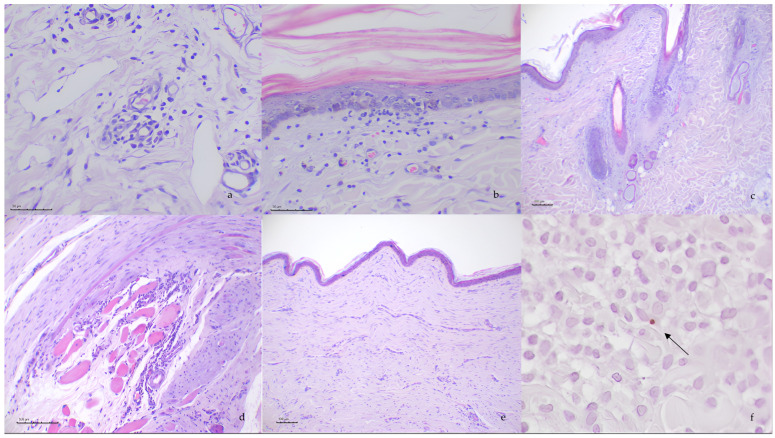
Photomicrographs of skin sections of ischemic dermatopathy with H&E: (**a**) lymphoplasmacytic perivascular to interstitial dermatitis, centered around dermal capillaries; (**b**) interface dermatitis composed of cell-poor infiltrates at the dermo–epithelial junction with the presence of intraepithelial lymphocytes in the epidermis; (**c**) follicular atrophy; (**d**) myositis composed of lymphoplasmacytic infiltrate between the muscle fibers; (**e**) dermal fibrosis and (**f**) immune-positive *Leishmania* spp. amastigote within a macrophage detected through immunochemistry (arrow).

Skin biopsies from all the patients demonstrated a similar lymphoplasmacytic and atrophic dermatitis composed of the following histopathological features: Mild to moderate lymphoplasmacytic perivascular to interstitial infiltrates was observed in all the cases (Figure 2a). Additionally, three of the cases had interface dermatitis characterized by minimal to mild superficial dermal inflammation that was oriented to the dermal–epidermal junction (Figure 2b), with degeneration of individual basal cells with apoptosis, hydropic degeneration and pigmentary incontinence. Diffuse pallor of the dermis characterized by collagen smudging and the increase in the connective tissue (Figure 2e) was reported in three of the patients. Hair follicles were severely atrophic or “faded” in all cases, and some of the atrophic follicles were replaced with amorphous material (Figure 2c). In the epidermis, three of the six dogs presented secondary ulceration with superficial crusting and edema and neutrophilic inflammation in the superficial dermis. Skeletal muscle was only available in the deeper samples in 4/6. Lymphoplasmacytic and—to a lesser extent—macrophagic and neutrophilic aggregates around muscle fibers and around vessels in the muscular tissue were observed in one case (Figure 2d). Moreover, one case presented similar inflammation of the piloerector muscles. Similar infiltrates were seen in the panicles of all the animals.

Mild vasculopathy was observed in the superficial and mild vascular plexuses in 4/6 dogs and was characterized by the combination of vasocongestion, endothelial cells appearing hypertrophied and plumbed into the vascular lumen (endothelial activation) and hemorrhagic foci. In three cases, more severe vascular damage was observed, consisting of capillary thrombosis, neutrophilic adhesion and migration, and in the arteries and arterioles, mild hypereosinophilia of the wall was observed (Table S2).

### 3.3. Histochemical and Immunohistochemical Evaluation

**Figure 3:**
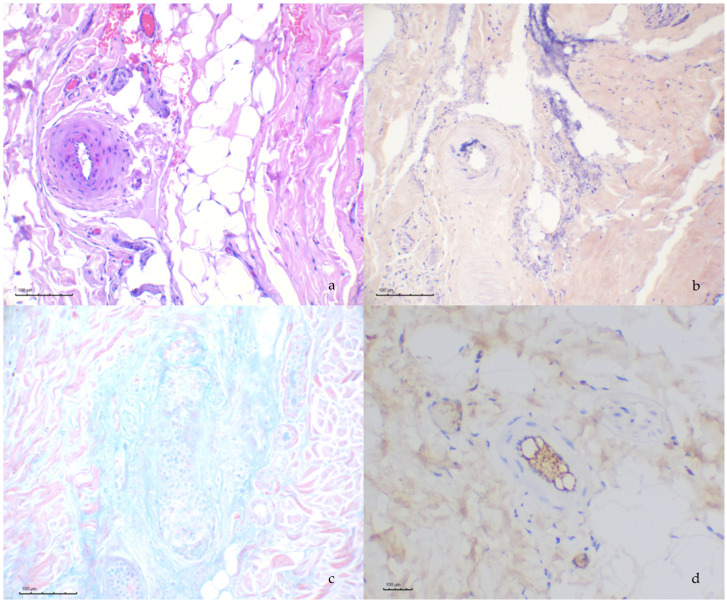
Histochemical and immunohistochemical findings: (**a**) a deep muscular artery displays mild eosinophilia of the tunica media; (**b**) the same artery reveals positive PTAH staining at the tunica media, consistent with fibrin; (**c**) abundant alcian blue positive material surrounding the hair follicle and (**d**) immunohistochemistry against IgG reveals positive staining in the lumen, albeit no labelling can be seen within the arterial wall.

Alcian blue stain revealed the mucinous change of the periadnexal collagen in 4/6 cases (Figure 3c). The affected arteries and arterioles evaluated through PAS stain failed to reveal proteinaceous material within the walls; however, in one case, fibrin was detected through PTAH stain within the walls of the affected arteries (Figure 3a,b).

Detection of IgG through immunohistochemical assay revealed intense signaling of the vascular lumina in the capillaries and—to a lesser extent—the arteries and veins in all cases. The capillary endothelial cells did not display positive staining. Absence of immunopositivity was also observed in the larger vessels in tunica intima, media and adventitia (Figure 3d).

### 3.4. Leishmania spp. Immunohistochemistry

The presence of *Leishmania* spp. in the skin sections was confirmed in 4/6 dogs (Figure 2f). *Leishmania* amastigotes were observed in macrophage inflammatory foci in the dermis, and their numbers were low in these four dogs.

## 4. Discussion

To the best of the authors’ knowledge, this is the first comprehensive description of ID in dogs with leishmaniosis.

Canine ID is a histopathological pattern characterized by microscopic skin lesions indicative of reduced blood flow. The histological characteristics of ID include follicular atrophy, lymphocyte-poor interface dermatitis, cell-poor vasculitis, dermal edema with mucin deposition and eosinophilic staining changes in the dermal collagen and vascular tunics, which give the collagen a “smudged” appearance [12]. Backel and collaborators described a detailed overview of the clinical presentation and treatment approaches of ID, excluding canine familial dermatomyositis [13]. In their study, other histological characteristics, such as vasculitis or vasculopathy (loss of vascular distinction or endothelial cells) and lymphoplasmacytic dermatitis or panniculitis, were described. In the present study, follicular atrophy and lymphoplasmacytic dermatitis were the most-reported changes in all the patients. Some details to highlight are that one of the dogs presented with myositis despite having no clinical signs associated with this inflammatory infiltrate in the musculature and that not all dogs presented obvious active vasculitis or vasculopathy. The presence of vasculitis and/or perivasculitis in ID is rather heterogeneous, and the cases studied also revealed a variable presence of vascular and perivascular infiltrates [13]. Whether or not vasculitis is observed in the microscopic skin sections depends mainly on the time at which the samples are taken and may also depend on the depth of the biopsies. Histopathological studies on familial dermatomyositis (a form of ID) revealed arteritis in muscular arteries [14,15]. Such vascular lesions were not observed in this study, while fibrinoid change was the main finding associated with larger vessels.

The pathogenesis of canine ID is largely unknown; it most likely relies on the deposition of CIC in dermal vessels. Although clinically similar, there may be different etiologies underlying this microscopic pattern. Typically, the development of cutaneous lesions is linked to triggering factors such as systemic infections, vaccines or drugs [5]. In the detailed overview of canine ID by Backel and collaborators in the USA, over half of the cases were considered to be generalized idiopathic and unlikely to be vaccine-associated, demonstrating the need to investigate other underlying causes of this condition. The presence of CIC in CanL has been well-documented [16], and the deposition of these immunocomplexes in tissues could potentially cause vasculitis, polyarthritis, uveitis, meningitis and glomerulonephritis [8]. Systemic necrotizing vasculitis has been observed in CanL, presenting with signs of blood vessel inflammation and ischemic damage across multiple organs and systems [17,18]. Unfortunately, although cutaneous lesions consistent with vasculitis—such as ulcers on the ear margins and tips, tail tip, nasal planum, footpads and claw beds with or without multifocal, scar-like alopecia due to ID— have been occasionally mentioned in case reports, a detailed or complete description is missing, and the underlying vasculopathy is rarely documented. This is likely because these lesions are infrequently biopsied due to their locations and because vascular damage tends to be transient and focal. In the present study, mild vasculopathy was observed in the superficial and mid-vascular plexuses in most dogs. However, more severe vascular damage was observed only in three dogs.

Even when histopathological lesions of vasculitis or vasculopathy are observed, as in our cases, confirming that *L. infantum* infection is the cause of vessel damage can be challenging. Direct diagnosis is not always possible, given the scarcity of macrophage infiltration in this histological pattern. In our case series, the presence of *Leishmania* spp. was confirmed by means of immunohistochemical tests only in four of the six dogs.

In a dog with cutaneous lesions suggestive of vascular damage, an indirect diagnosis of the causative role of *Leishmania* can be accomplished through the demonstration of elevated levels of anti-*Leishmania* antibodies. In addition to canine leishmaniosis, immune complexes are believed to play an important role in other canine vector-borne diseases such as ehrlichiosis or dirofilariosis. For this reason, all patients included in this study were required to test negative for this group of diseases as an inclusion criterion [19,20]. It is interesting to note that the patients presented in this case series manifested clinical signs suggestive of ID simultaneously with the presence of elevated antibody levels before treatment or during the first month post-treatment. It is well known that, although antileishmanial antibody levels begin to decrease after treatment, they remain elevated for the first 6 months post-treatment [21].

A positive response to antileishmanial treatment may help to confirm or rule out the role of *Leishmania* in ID. In our case series, only one dog with negative immunohistochemistry did not show a favorable response. It should be highlighted that the response to treatment can be only partial in ID, even when leishmaniosis is the underlying cause, because hair regrowth may not occur despite antileishmanial treatment if follicular atrophy is irreversible.

Assessment of IgG within tissues through immunohistochemistry has not been yet attempted in canine ID. Immunohistochemistry against IgG is helpful in the diagnosis of several immune-mediated diseases [22]. IgG positive immunostaining in the lumen of blood vessels represents the detection of circulating IgG and is always expected; however, in the present study, the IgG signal was not detected in vessel walls. Absence of IgG-positive labelling could be explained by suboptimal technique sensitivity or the clearing of immune complexes by the time of sampling. Alternatively, immune complexes could be present only within endothelial cells, which would mask these complexes due to the fact that the intense luminal labelling of these cells presents as a normal finding.

The limitations of our study include the following: (i) the small number of cases analyzed; (ii) the retrospective nature of the study, which resulted in the unknown and variable timing of skin sampling during the course of the disease; and (iii) the previously mentioned challenges related to IgG immunostaining.

Further investigation, including case-control studies currently underway, is required to clarify this potential link and to deepen our understanding of the pathogenesis.

## 5. Conclusions

In conclusion, ID can be associated with *L. infantum* infection in dogs. The clinical and histopathological features observed in this study closely resemble those previously reported in ID of other etiologies. While the exact causal relationship remains uncertain, we hypothesized that the high levels of circulating anti-*Leishmania* antibodies would be at the bases that leishmaniosis may be an underlying factor contributing to ID development.

## Data Availability

The original contributions presented in this study are included in the article/Supplementary Materials. Further inquiries can be directed to the corresponding author.

## Supplementary Materials

The following supporting information can be downloaded at: https://www.mdpi.com/article/10.3390/pathogens14030246/s1, Table S1: Clinical information of dogs with ID and leishmaniosis, Table S2: Histopathological findings.

## Abbreviations

The following abbreviations are used in this manuscript:

ID	Ischemic dermatopathy.
CanL	Canine leishmaniosis.
CIC	Circulating immune complexes.
PAS	Periodic acid–Schiff.
PTAH	Phosphotungstic acid–haematoxylin.
UPC	Urinary protein creatinine ratio.
H&E	Hematoxylin and eosin.
