# Supplementary material for: Clinical, Immunological and Pathological Characteristics of Ischemic Dermatopathy in Dogs with Leishmaniosis
Source: Pathogens. 2025 Mar 3;14(3):246. doi: 10.3390/pathogens14030246 (PMC11944567; doi:10.3390/pathogens14030246)
Supplement: Supplementary file 1 [file pathogens-14-00246-s001.zip › pathogens-3483604-supplementary.pdf]

Table S1. Clinical information of dogs with ID and leishmaniosis.

Case	Breed, age (years) and sex	Lesions	Time of onset	Response to treatment during three months after diagnosis
1.	Hound, 8, M	Multifocal alopecia, onychodystrophy	During the first month of anti-*Leishmania* treatment	NR
2.	Chihuahua, 5, F	Multifocal alopecia, ulcers	At the time of diagnosis of leishmaniosis	GR
3.	Whipped, 4, M	Multifocal alopecia	At the time of diagnosis of leishmaniosis	GR
4.	Mixed breed, 2, M	Multifocal alopecia, onychodystrophy, ulcers	At the time of diagnosis of leishmaniosis	GR
5.	American Bully, 2, M	Multifocal alopecia	During the first month of anti-*Leishmania* treatment	GR
6.	Greyhound, 5, F	Multifocal alopecia, ulcers	At the time of diagnosis of leishmaniosis	GR
7.	Greyhound, X, F	Multifocal alopecia	At the time of diagnosis of leishmaniosis	GR

M: male, F: female, NR: nonresponse, GR: good response.

Table S2. Histopathological findings

**Case**	**1**	**2**	**3**	**4**	**5**	**6**	**7**
Lymphoplasmacytic perivascular to interstitial dermatitis	Yes	Yes	Yes	Yes	Yes	Yes	Yes
Follicular atrophy	Yes	Yes	Yes	Yes	Yes	Yes	Yes
Ulceration	Yes	Yes	No	Yes	No	No	No
Interface dermatitis	Yes	Yes	No	Yes	No	No	Yes
Changes in the dermal collagen	Yes	Yes	Yes	Yes	Yes	Yes	No
Vasculopathy	Yes	Yes	Yes	Yes	No	No	Yes
Panniculitis	Yes	Yes	Yes	Yes	Yes	Yes	Yes
Myositis	No	No	Yes	Yes	No	No	Yes
